# The Role of [^18^F]FDG-PET/CT in Predicting Malignant Transformation of Plexiform Neurofibromas in Neurofibromatosis-1

**DOI:** 10.1155/2016/6162182

**Published:** 2016-12-12

**Authors:** David Tovmassian, Muzib Abdul Razak, Kevin London

**Affiliations:** ^1^Westmead Hospital, Cnr Hawkesbury and Darcy Rd, Westmead, NSW 2145, Australia; ^2^University of Sydney, Camperdown, NSW, Australia; ^3^Nuclear Medicine, Westmead Children's Hospital, Westmead, NSW, Australia

## Abstract

*Background*. Malignant peripheral nerve sheath tumours (MPNSTs) are difficult to diagnose and treat and contribute to significant morbidity and mortality for patients with Neurofibromatosis-1 (NF-1). FDG-PET/CT is being increasingly used as an imaging modality to discriminate between benign and malignant plexiform neurofibromas.* Objectives*. To assess the value of FDG-PET/CT in differentiating between benign and malignant peripheral nerve lesions for patients with Neurofibromatosis-1.* Methods*. A systematic review of the literature was performed prior to application of stringent selection criteria. Ultimately 13 articles with 796 tumours were deemed eligible for inclusion into the review.* Results*. There was a significant difference between mean SUV_max_ of benign and malignant lesions (1.93 versus 7.48, resp.). Sensitivity ranged from 89 to 100% and specificity from 72 to 94%. ROC analysis was performed to maximise sensitivity and specificity of SUV_max_ cut-off; however no clear value was identified (range 3.1–6.1). Significant overlap was found between the SUV_max_ of benign and malignant lesions making differentiation of lesions difficult. Many of the studies suffered from having a small cohort and from not providing histological data on all lesions which underwent FDG-PET/CT.* Conclusion*. This systematic review is able to demonstrate that FDG-PET/CT is a useful noninvasive test for discriminating between benign and malignant lesions but has limitations and requires further prospective trials.

## 1. Introduction

Neurofibromatosis type one (NF-1) is a common inherited disorder affecting from 1 in 2,000 to 1 in 5,000 live births; it is an autosomal dominant condition characterized by cutaneous lesions, skeletal dysplasias, and the tendency to form soft tissue tumours on peripheral nerves such as plexiform neurofibromas. These plexiform neurofibromas have potential for sarcomatous transformation to malignant peripheral nerve sheath tumours (MPNSTs) [1, 2]. MPNSTs represent 5–10% of all soft tissue sarcomas and are more prevalent in NF-1, contributing significantly to the morbidity and mortality of these patients. MPNSTs carry an ominous prognosis with a 5-year survival of up to 60% due to delayed diagnosis, early metastasis, and poor response to systemic therapy [1, 3].

The mainstay of treatment for MPNSTs remains surgical excision. The ability for benign neurofibromas to mimic MPNST with clinical features such as increased growth rate, irregular contour, and pain has made diagnosis without surgical excision for histology challenging. Contrast enhanced CT and MRI have been shown to be a suboptimal imaging modality for diagnosis of potential MPNST in NF-1; despite being helpful in detecting nodular lesions these imaging modalities have variable potential in differentiating between benign and malignant disease and limited ability to quantitatively analyse suspicious lesions [4–6].

[^18^F]2-Fluoro-2-deoxy-D-glucose PET/CT (FDG-PET/CT) is an imaging modality that noninvasively assesses in vivo glucose metabolism and is commonly used to stage and monitor treatment response and investigate for recurrence in solid tumour malignancies. A maximal standardized uptake value (SUV_max_) is a unitless semiquantitative measure of FDG uptake and is used to assess the metabolic activity within a potentially malignant tumour. The use FDG-PET/CT for the diagnosis of MPNST in patients with NF-1 has been a key area of research. Establishing a noninvasive way of diagnosing MPNSTs may lead to earlier treatment and improved prognosis. The use of tissue histopathology to establish a definitive diagnosis is highly specific but may require complete surgical excision of a suspect tumour which carries increased morbidity and mortality to the patient as well as a technical challenge.

There have been multiple published studies assessing the use of semiquantitative FDG-PET/CT analysis by calculating the maximal standardized uptake value (SUV_max_) within a tumour to differentiate between benign and malignant peripheral nerve sheath tumours with varying results. The purpose of this systematic review is to synthesise and appraise the current evidence on the role of FDG-PET/CT in diagnosing MPNST as well as potential future research directions.

## 2. Materials and Methods

### 2.1. Study Selection and Eligibility Criteria

A review of the English language articles on online databases PubMed, MEDLINE, Embase, and Scopus was performed using MeSH/key terms “Nerve Sheath Neoplasms”, “Positron-Emission Tomography”, “Neurofibromatosis 1”, and “Peripheral nerve sheath tumors”.

**Figure 1:**
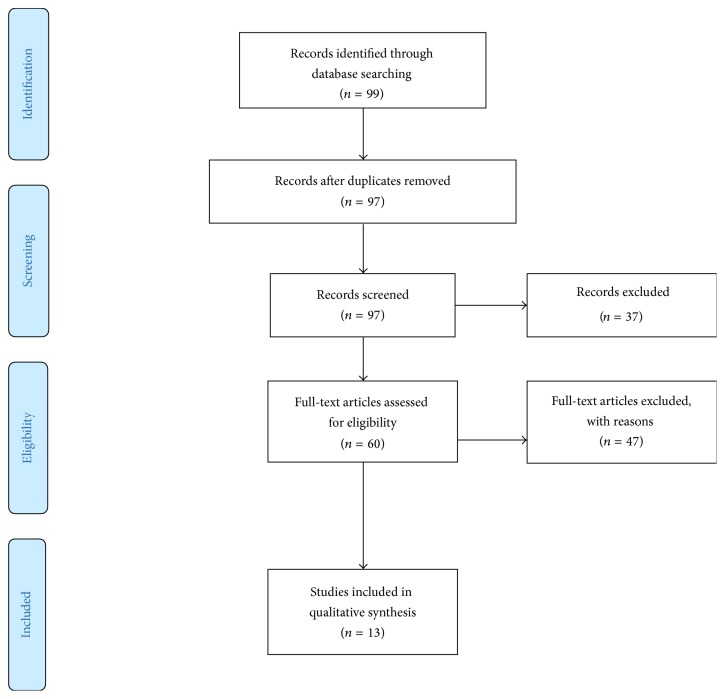
Study selection and search strategy.

Eligible publications were required to have included SUV_max_ for the semiquantitative analysis of plexiform neurofibromas to diagnose malignant transformation with the reference standard as histopathological correlation or informed clinical follow-up. Articles focusing primarily on the use of other quantitative variables such as the tumour-to-liver ratio were not included. Case reports, conference abstracts, posters presentations, book chapters, and review articles were excluded from this review.  Figure 1 describes the study selection process.

### 2.2. Synthesis of Results

Qualitative analysis of the studies was performed with contribution from each study's data items of interest. A meta-analysis was not performed due to a lack of sufficient homogeneity between the studies. Data was extracted using a predetermined standardized table.

## 3. Results

A total of 97 articles were found once duplicates were removed, the abstracts of these articles were reviewed and 23 articles were excluded due to being small case reports; 14 articles were excluded due to being review articles, book chapters, abstracts for presentation, and posters or pertaining to veterinary science. 60 full-text articles were then reviewed for relevance to our clinical question; further 47 articles were excluded with 3 papers containing duplicate data. Review of the citations for all relevant studies did not yield any further trials. Ultimately 13 articles were eligible for this review; there were no randomised control trials identified for this review.  Table 1 depicts the demographics of included studies.

### 3.1. Imaging Modalities

Included studies all had patients administered intravenous [^18^F]2-fluoro-2-deoxy-D-glucose following a fasting time of 4–6 hours. Whole body PET with CT imaging was then acquired. The interval between administration of FDG and acquisition of imaging varied slightly, early images were taken between 45 and 90 minutes after administration, and for articles which included delayed imaging this was performed 240 minutes after administration of FDG.

### 3.2. Diagnostic Potential of FDG-PET/CT

Table 2 shows characteristics of the included studies regarding their SUV_max_ of malignant and benign lesions as well as the sensitivity, specificity, positive predictive value, negative predictive value, and accuracy. Mean SUV_max_ was 1.93 and 7.48 for benign and malignant lesions, respectively, across all trials.

The medians for PPV and NPV were 40% and 100%, respectively, across the studies with a mean accuracy of 83.5%.

### 3.3. Optimum SUV_max_ Cut-Off

Optimum SUV_max_ to maximise sensitivity and specificity is shown in Table 2. A wide range is noted. Sensitivities ranged from 91% to 100% although specificity ranged from 72% to 95%.

ROC analysis was performed in selected studies for optimum SUV_max_ cut-off [12, 13, 16, 18, 19]. These values yielded cut-offs of 3.1, 3.2, 3.5, 4.1, and 6.1 to achieve maximum sensitivity and specificity of identifying malignant lesions.

### 3.4. Primary and Secondary Outcomes

There is a noted difference in the SUV_max_ of benign versus malignant plexiform neurofibromas as shown in Table 2; there is however considerable overlap in ranges of SUV_max_ for benign and malignant lesions. FDG-PET/CT was shown to be effective in the diagnosis of malignant lesions with the mean sensitivity of 91%. There was insufficient evidence to accept a universal cut-off value for SUV_max_ (ROC cut-off ranging from 3.1 to 6.1) which is reinforced with the aforementioned range in specificity.

### 3.5. Comparative Data

There is difficulty to directly compare these studies due to lack of sufficient homogeneity. Two of the included trials focus on the paediatric population [14, 15]. Table 2 demonstrates the variable FDG uptake time prior to imaging being performed. None of the studies stratified patients based upon having previous surgery or systemic therapy.

## 4. Discussion

### 4.1. Diagnostic Benefit of FDG-PET/CT

Ferner et al.'s [7] article showed that quantitative analysis malignant lesions had a statistically significant increase in SUV_max_ compared to benign lesions (mean 5.4 ± 2.4 versus 1.54 ± 0.7, resp., *p* = 0.002). This finding of a statistically significant difference in means was also evident in articles published by Cardona et al. and Bredella et al. [8, 9]. Qualitatively 2 benign tumours were classified as malignant; however there were no false negatives and an overlap of SUV_max_ readings for benign and malignant tumours was identified between SUV_max_ 2.7 and 3.3 by Ferner et al. [7]. This range of overlapping SUV_max_ values between benign and malignant lesions remains the greatest issue with FDG-PET/CT in this context. Tumours which present as a false positive tend to be within this overlapping region as evidenced by Table 2 that malignant lesions will likely have a much higher SUV_max_ on average.

In 2008 Ferner et al. [10] performed a follow-up trial that included FDG-PET/CT with 4-hour delay from injection of tracer to proceeding with imaging as this was determined to be the optimal time in their previous study [7]. Mean SUV_max_ of benign and malignant lesions were 1.5 and 5.7, respectively. No malignant tumours were found with SUV < 1.5; however there were 3 benign tumours with SUV > 3.5. Between SUV_max_ 2.5 and 3.5 seven benign and six malignant lesions were found. There were four false positive and three false negative scans. The sensitivity for high-grade MPNSTs was 100%. The use of delayed imaging with 4-hour delay has shown some potential; Ferner et al. [10], Warbey et al. [12], and Chirindel et al. [19] all included delayed imaging 4 hours after FDG administration. Warbey et al. [12] were able to demonstrate a statistically significant difference between early and delayed imaging for lesions classified on FDG-PET/CT (*p* = 0.002) as malignant but not for benign lesions. The mean SUV_max_ for malignant lesions increased from 7.0 on early to 8.1 on delayed imaging; they were able to obtain sensitivity and specificity of 97% and 87%, respectively. Chirindel et al. [19] were unable to replicate these results however having 84% specificity for early versus 81% for delayed imaging.

Benz et al. [13] in 2010 did a combined prospective and retrospective study mean SUV_max_ for MPNSTs which was found to be significantly higher than benign lesions (12.0 + 7.1 versus 3.4 + 1.8, *p* < 0.001). There were two false positive and one false negative scans in this cohort. ROC analysis concluded an optimal SUV_max_ cut-off of 6.1 leading to sensitivity and specificity of 94% and 91%, respectively; this is significantly higher than thresholds that were determined from studies by Warbey et al., Derlin et al., Salamon et al., and Chirindel et al. [12, 16, 18, 19] which would lead to several false negative scans. PPV, NPV, and diagnostic accuracy were 89%, 95%, and 93% (*p* < 0.001), respectively.

Moharir et al. [14] retrospectively analysed 18 children with NF-1 who underwent plexiform neurofibroma surveillance and revealed a sensitivity and a specificity of 100% and 86%, respectively, with a PPV of 50% and NPV of 100%. This is the first study to evaluate the utility of FDG-PET/CT for children and concludes that although in this trial they used only early imaging 45 minutes after FDG injection they go on to state that early and delayed imaging are now their standard practice due to the findings of Warbey et al. [12]. In addition, Tsai et al. [15] also analysed the paediatric population and found that the mean SUV_max_ of benign and malignant lesions were 2.49 and 7.63, respectively. Using SUV_max_ cut-offs of 3, 4, and 5 yielded a sensitivity of 100%, 100%, and 89% and a specificity of 81%, 94%, and 94%, respectively. Eight of 27 lesions were MPNST and none had SUV_max_ < 4. Of the 16 plexiform neurofibromas 8 were classified as atypical, that is, with histological findings consisting of hypercellularity and hyperchromatic nuclei with the absence of mitotic figures [20]; one of these lesions had SUV_max_ of 6.90. Although atypical neurofibromas can make histological diagnosis difficult, they are classified as benign; they can transform to MPNST; however plexiform neurofibromas also have this ability and either can display varying SUV_max_. There exists a significant overlap between plexiform neurofibromas, atypical neurofibromas, and MPNSTs on FDG-PET/CT. These findings have obvious correlation with the adult population; however the sample sizes remain small and therefore it becomes difficult to validate these findings. Important to note is that not all lesions with overlapping SUV_max_ are found atypical; a range of SUV_max_ can be associated regardless of histological diagnosis.

SUV_max_ is calculated by dividing the activity concentration within the tissue by the injected activity/body size. There are several factors that can affect the SUV_max_ measurement including biological factors such as body weight/size, blood glucose level, respiratory effort, and the amount of time between injection of radionuclide and scanning. Technical factors can also impact on SUV_max_ such as scanner variability, reconstruction parameters, calibration error, and interuser variability [21]. A trial by Velasquez et al. [22] to determine the reproducibility of SUV_max_ findings in patients with scans taken 7 days apart was able to produce a coefficient of variability of 10%–12% which increased up to 21% when variables such as time from injection to scan were changed.

Salamon et al. [18] provided values for TTL ratio which may provide, in addition to SUV_max_, a potential method to decrease aforementioned variability by referencing the patient's own tissue uptake of FDG. Salamon et al. [18] were able to show a statistically significant difference in mean SUV_max_ between benign and malignant lesions using the established cut-off of 3.5; they were able to increase specificity from 64.5% using SUV_max_ to 90.3% with TTL ratio [18]. These findings of increasing specificity with the incorporation of TTL have been reproduced; however more data is required in order to impact clinical decision making.

### 4.2. Optimal SUV_max_ Cut-Off

No optimal SUV_max_ cut-off exists in the published literature. The use of SUV_max_ 3.5 as a cut-off was adopted by some trials [16, 18]. ROC analysis was performed as noted in Table 2 which shows the optimum SUV_max_ cut-off to maximise sensitivity and specificity. The range varies; however all but one fall between 3.0 and 4.0. This makes interpretation difficult as this level is the grey zone within which both benign and malignant lesions can occur. Further research is needed with addition of delayed imaging in order to better guide clinical decision making.

### 4.3. Novel Parameters

This paper looks exclusively at the value of SUV_max_ for distinguishing benign and malignant disease, as aforementioned SUV_max_ has its own limitations as a semiquantitative method of analysis and the use of novel parameters may be able to eliminate some of this variability. Novel parameters such as metabolic tumour volume (MTV) and total lesion glycolysis (TLG) have been used which have shown promise but lack adequate evidence to justify routine use. Two retrospective studies were identified showing with statistical significance (*p* < 0.01) that on patient and lesion basis MPNSTs had a higher rate of metabolic tumour volume (MTV) and total lesion glycolysis (TLG) [23, 24]. A further trial was found showing that TLG was a useful prognostic marker when compared to SUV_max_, TTL ratio, and HI_max_ [25]. This allows avenues for future research to validate the utility of these novel parameters in the assessment of patients with NF-1; currently there is only sparse literature with evidence of selection bias for its role in distinguishing between benign and malignant lesions. Derlin et al. [16] provided values in addition to SUV_max_, namely, with a Homogeneity Index SUV—incorporating homogeneity of the lesion. They were able to demonstrate a statistically significant increase in specificity between benign and malignant lesions with HI_max_ which provides avenue for further research [16].

It is clear that there is no single ideal method or parameter for noninvasively distinguishing between benign and malignant disease in this cohort of patients. The use of FDG-PET/CT and its various parameters such as SUV_max_, TTL ratio, MTV, TLG, and other imaging techniques such as contrast enhanced CT and MRI must be used along with clinical findings for individual patients. SUV_max_ does remain the single best parameter available currently with the most support in the literature; this may change in the future with ongoing research.

### 4.4. Bias and Study Designs

There are certain bias and study designs which must be noted in this systematic review. Studies tended to lean towards patients who had already gone on to have histological analysis meaning they may have had more clinically advanced disease. Consideration must be given to the small sample sizes in the majority of the trials included which makes them much more prone to Type I error. Additionally despite selection bias for patients who have had histological analysis there still exists a large amount of patients whose results are based purely on clinical follow-up which may not provide adequate information. Additionally studies introducing new units of measurement of FDG uptake have not been validated and the results are therefore difficult to interpret.

## 5. Conclusion

In summary, MPNST is a life-threatening disease that is known to transform from previously benign lesions in patients with NF-1. FDG-PET/CT has been shown to be a useful, noninvasive diagnostic tool for the assessment of malignant transformation of PNSTs in adults and children. It is able to predict with excellent sensitivity and negative predictive value whether malignant transformation has occurred. It does however have shortcomings in that there is no ideal SUV_max_ cut-off value that has been found and substantiated; although multiple trials have used 3.5 as a cut-off there continues to be several false positive lesions [12, 16, 18].

The use of delayed imaging has a role in being able to reduce the number of false positive findings; however this has been shown to have technical restraints and would require further trials to validate findings. The use of a normalised SUV whether to the patient's liver uptake or lean body mass such as that performed by Salamon et al. and Chirindel et al. does also have a potential role in differentiating between benign and malignant PNSTs; however the data on this is limited [18, 19].

Further prospective trials are required in order to establish an ideal SUV_max_ cut-off, to determine the use of tumour-to-liver ratio and other normalised values, and to increase the pool of data available in this area and should be performed in a uniform fashion.

## Figures and Tables

**Table 1:** Basic demographic characteristics of included studies.

Authors	Year	Patients (*n*)	Tumours analysed (*n*)	Gender (% male)	Mean age (years)
Ferner et al. [7]	2000	18	23	44	28
Cardona et al. [8]	2003	13	25	38	46
Bredella et al. [9]	2007	45	50	49	37
Ferner et al. [10]	2008	105	116	49	31
Karabatsou et al. [11]	2009	9	9	56	38
Warbey et al. [12]	2009	69	85	*50*	31
Benz et al. [13]	2010	34	40 (14 NF1)	59	46
Moharir et al. [14]	2010	18	16	33	9
Tsai et al. [15]	2012	20	27	35	15
Derlin et al. [16]	2013	31	99	42	30
Meany et al. [17]	2013	15	61	53	18
Salamon et al. [18]	2014	50	152	41	33
Chirindel et al. [19]	2015	41	93	34	36

**Table 2:** Characteristics of the FDG-PET/CT studies.

Authors	Time between oral contrast administration and scan (minutes)	Mean SUV_max_ of benign lesions	Mean SUV_max_ of malignant lesions	Sensitivity (%)	Specificity (%)	Positive predictive value (%)	Negative predictive value (%)	Accuracy (%)	ROC analysis optimal SUV_max_ cut-off
Ferner et al. [7]	55–60	1.54 ± 0.7	5.4 ± 2.4	n/a	n/a	n/a	n/a	n/a	n/a
Cardona et al. [8]	n/a	1.0	4.1	100	83	n/a	n/a	n/a	n/a
Bredella et al. [9]	45–60	1.5	8.5	95	72	71	95	82	n/a
Ferner et al. [10]	240	1.5 ± 1.06	5.7 ± 2.6	89	95	n/a	n/a	n/a	n/a
Karabatsou et al. [11]	60	2.6	10.4	n/a	n/a	n/a	n/a	n/a	n/a
Warbey et al. [12]	90 and 240	2 (1.9)^a^	7 (8.1)^a^	97	87	n/a	n/a	n/a	3.1
Benz et al. [13]	60	2.3 ± 0.7	12.8 ± 8.6	94	91	n/a	n/a	93	6.1
Moharir et al. [14]	45	n/a	n/a	100	86	50	100	n/a	n/a
Tsai et al. [15]	60	2.49 ± 1.5	7.63 ± 2.96	100/100/89^b^	81/94/94^b^	n/a	n/a	n/a	n/a
Derlin et al. [16]	60	1.7 ± 0.5	5.6 ± 2.7	100	74	28	100	77	4.1
Meany et al. [17]	60–90	n/a	n/a	n/a	n/a	n/a	n/a	n/a	n/a
Salamon et al. [18]	n/a	2.56	8.61	100	79.8	40	100	82	3.5
Chirindel et al. [19]	60 and 240	2 (2.3)^a^	6.5 (8.3)^a^	91	84 (81)^a^	67 (63)^a^	96	n/a	3.2

n/a: not available.

^a^Value for delayed imaging.

^b^Values as per SUV_max_ cut-off of 3/4/5.

## Additional Points

We systematically review the data on the use of FDG-PET/CT for determining malignant transformation of plexiform neurofibromas. FDG-PET/CT although imperfect provides extremely useful information for the management of patients with NF-1 and clinical signs of malignancy.
